# Experimental study and clinical significance of the impact of gastrointestinal motility disorders on hepatic blood perfusion

**DOI:** 10.3389/fmed.2025.1643351

**Published:** 2025-09-25

**Authors:** Jin Tonghui, Liu Tiejun, Liu Chaoyue, Wang Han

**Affiliations:** ^1^Changchun University of Chinese Medicine, Changchun, Jilin, China; ^2^Affiliated Hospital of Changchun University of Chinese Medicine, Changchun, Jilin, China; ^3^China Academy of Chinese Medical Sciences, Beijing, China

**Keywords:** gastrointestinal motility disorders, hepatic blood perfusion, portal vein blood flow, liver function, clinical prognosis

## Abstract

**Introduction:**

The gastrointestinal tract and liver maintain intricate hemodynamic relationships through the portal venous system. While gastrointestinal motility disorders are known to affect digestive function, their impact on hepatic blood perfusion remains poorly understood—particularly in the context of hepato-gastrointestinal interactions—limiting comprehensive patient management approaches. This study aimed to investigate the mechanism and clinical significance of the impact of gastrointestinal motility disorders on hepatic blood perfusion, providing a theoretical basis for the diagnosis and treatment of related diseases.

**Methods:**

Sixty patients with gastrointestinal motility disorders, admitted from January 2023 to December 2024, were selected as the study group, with 60 healthy individuals during the same period forming the control group. Hepatic blood perfusion status was evaluated through abdominal ultrasound Doppler and CT perfusion imaging, while gastrointestinal motility function was assessed using electrogastrography and gastrointestinal motility monitoring systems.

**Results:**

Portal vein flow velocity, hepatic arterial blood flow, and hepatic perfusion index in patients with gastrointestinal motility disorders were significantly lower than those of individuals in the control group (*p* < 0.05). The degree of gastrointestinal motility disorder showed a significant negative correlation with hepatic perfusion parameters (*r* = −0.681, *p* < 0.01), with clinically significant thresholds identified for intervention planning.

**Discussion:**

Gastrointestinal motility disorders can significantly affect hepatic blood perfusion status. Clinically, attention should be paid to changes in liver function in patients with these disorders, focusing on early intervention to improve prognosis. These findings have important implications for clinical monitoring protocols and treatment strategies.

## 1 Introduction

Recent international studies have highlighted the complex relationships between gastrointestinal dysfunction and hepatic complications, yet the specific mechanistic pathways remain inadequately characterized (1–3). The gastrointestinal tract and liver maintain a close and complex relationship in the context of blood circulation (4–6). The portal venous system serves as an important bridge connecting these two organs, carrying venous return blood from the stomach, small intestine, large intestine, pancreas, and spleen (7, 8). This blood is rich in nutrients and metabolic products, and flows directly into the liver for further biochemical processing (9). The liver receives precise regulation from a dual blood supply system, with the portal vein providing approximately 75% of blood flow—primarily responsible for nutrient transport—while the hepatic artery provides the remaining 25% of blood flow (10, 11), ensuring adequate oxygenated blood supply to liver tissue.

International consensus defines gastrointestinal motility disorders as functional abnormalities affecting 10–40% of the global population, yet their systemic consequences beyond digestive symptoms remain poorly understood (12, 13). The mechanism involved in the regulation of hepatic blood perfusion involves multiple levels of physiological control systems, including the hepatic arterial buffer response, portal venous pressure regulation, release of vasoactive substances, and coordinated actions of the neuroendocrine system (14). These mechanisms collectively maintain the relative stability of hepatic blood flow and orchestrate adaptive responses to changes.

Gastrointestinal motility disorders, as common functional diseases of the digestive system, are characterized by abnormal gastric and intestinal smooth muscle contraction rhythms, weakened peristaltic function, and decreased digestive tract transport capacity (15–17). This pathological state not only affects digestive function but may also significantly impact hepatic blood perfusion by altering gastrointestinal blood circulation patterns (18).

Currently, the specific quantitative relationship between gastrointestinal motility disorders and alterations in hepatic perfusion remains poorly understood, hindering the development of evidence-based clinical strategies. Addressing a critical knowledge gap, this study establishes quantitative relationships between motility dysfunction and hepatic perfusion, with potential implications for clinical practice guidelines. In-depth exploration of the interrelationship between gastrointestinal motility disorders and hepatic blood perfusion holds important theoretical value and guiding significance for understanding the pathophysiological mechanisms underlying diseases of the digestive system.

## 2 Materials and methods

### 2.1 Study subjects

Patients with gastrointestinal motility disorders who visited the gastroenterology outpatient department and inpatient ward of the Affiliated Hospital of Changchun University of Chinese Medicine from January 2023 to December 2024 were selected as the study group.

Inclusion criteria were as follows: (1) Fulfilment of the diagnostic criteria of the “Chinese Guidelines for Diagnosis and Treatment of Gastrointestinal Motility Disorders,” aligned with international Rome IV criteria for standardization; (2) Age 18–75 years, with disease duration exceeding 3 months and stable symptoms; (3) Provision of informed consent and signed consent forms by the patients and their families; and (4) Ability to cooperate with completion of relevant examination items.

Exclusion criteria were as follows: (1) Presence of severe cardiopulmonary diseases, hepatic or renal insufficiency, or malignant tumors, including baseline liver dysfunction (ALT/AST >2 × upper normal limit) to ensure hepatic perfusion measurements reflect motility-related changes; (2) Pregnant and/or lactating status, presence of psychiatric diseases resulting in inability to cooperate with examinations; (3) Recent use of medications affecting gastrointestinal motility or hepatic blood flow (beta-blockers, nitrates) within 72 h of assessment; (4) History of abdominal surgery that might affect examination results.

Healthy individuals undergoing physical examinations during the same period were selected as controls if they had normal liver function, no history of digestive system diseases, and no abnormalities on imaging examinations.

The gastrointestinal motility scoring scale was used to assess the severity of gastrointestinal motility disorders (19, 20). Ensuring balanced distribution of sample sizes across groups to provide reliable data foundation for subsequent statistical analysis, the cases were grouped as follows: (1) Mild group scored 8–15 points on the scale; moderate group, 16–23 points; and severe group, 24–30 points.

### 2.2 Data collection and processing

A standardized clinical data collection form was established to ensure completeness and accuracy of data collection.

The following general clinical data were collected: (1) Patients’ sex, age, height, weight, occupation, education level, and other basic information; (2) Vital signs including blood pressure, heart rate, body temperature, respiratory rate, and other physiological parameters; and (3) Patients’ lifestyle habits such as smoking, alcohol consumption, and dietary structure that might affect study results.

The following information on medical history and comorbidities was recorded: (1) Detailed history of diseases of the digestive system, surgical history, drug allergy history, family genetic disease history, and other relevant medical information; (2) Diseases that might affect gastrointestinal motility, such as diabetes, thyroid dysfunction, and neurological diseases; and (3) Previous hospitalization treatment experiences and efficacy evaluations.

Medication history was recorded as follows: (1) All currently used medications including names, dosages, duration of use, and administration methods; (2) Focus on gastrointestinal motility regulators, anticholinergic drugs, opioid analgesics, and other medications that might affect study results; and (3) Patient medication compliance and adverse reaction occurrences.

### 2.3 Research methods

Multiple advanced detection technologies were employed to comprehensively evaluate patients, ensuring scientific reliability of research data.

Gastrointestinal motility function was assessed as follows: (1) Multi-channel electrogastrography monitoring system was used (Star Medical EGG-3D, frequency range 0.5–9.0 cpm, sampling rate 1 Hz); patients were examined after 12 h of fasting, recording parameters such as frequency, amplitude, and rhythmicity of gastric electrical activity. Gastric electrical rhythm disorder rate was defined as the percentage of abnormal slow wave activity outside the normal frequency range (2.5–3.7 cycles/min). The recording duration was standardized at 30 min in the fasting state followed by 60 min post prandium after a standardized 520-kcal test meal (composition: 15% protein, 25% fat, 60% carbohydrate); (2) Gastric emptying scintigraphy (99mTc-sulfur colloid labeled solid meal, imaging at 0, 1, 2, and 4 h post-ingestion using dual-head gamma camera) was performed to measure gastric emptying time and evaluate gastric antral contractile function as well as gastric content transport capacity; (3) Small-bowel transit time measurement was performed (lactulose hydrogen breath test with breath samples collected every 15 min for 3 h) to assess overall gastrointestinal motility function status.

The following hepatic blood perfusion detection techniques were applied: (1) High-resolution color Doppler ultrasound was used (Philips EPIQ 7, C5-1 curved array transducer, 2–5 MHz) to measure hemodynamic parameters such as portal vein flow velocity, hepatic arterial resistance index, and hepatic arterial peak flow velocity (21), following standardized protocols established in international hepatic imaging studies. Portal vein measurements were obtained at the main portal vein trunk level with sample volume adjusted to 2–3 mm, angle of insonation <60°, and measurements averaged over 5 cardiac cycles; (2) CT perfusion imaging technology was employed (Siemens SOMATOM Force, 80kVp, 150mAs, 5 mm slice thickness, contrast agent: 1.5 mL/kg iohexol 350 mgI/ml at 5 mL/s, followed by 20 mL saline flush) for quantitative analysis of perfusion parameters such as hepatic blood flow, blood volume, mean transit time, and hepatic perfusion index (22), with hepatic perfusion index (HPI) calculated using validated software algorithms. ROI placement was standardized at the right hepatic lobe (segments V–VIII), avoiding vessels and bile ducts, with minimum ROI size of 1 cm^2^. Images were acquired every 2 s for 40 s during the first pass; (3) Magnetic resonance perfusion imaging was used as a supplementary examination method to improve detection accuracy (1.5 T MRI, gadolinium-DTPA 0.1 mmol/kg, dynamic imaging every 3 s for 3 min).

Measurement of liver function indicators was carried out as follows: (1) Detection of liver enzyme indicators such as serum alanine aminotransferase, aspartate aminotransferase, total bilirubin, and direct bilirubin; (2) Measurement of liver synthetic function indicators such as albumin, globulin, and prothrombin time; (3) Automated biochemical analyzers were used for detection, with all examinations performed by the same group of experienced technicians.

### 2.4 Observation indicators

A comprehensive indicator monitoring system was established to comprehensively evaluate the impact of gastrointestinal motility disorders on hepatic blood perfusion.

The following primary evaluation indicators were selected based on their demonstrated clinical relevance in international literature: (1) Core hemodynamic parameters such as portal vein flow velocity, hepatic arterial blood flow, HPI, and total hepatic blood flow; (2) Quantitative parameters from CT perfusion imaging such as hepatic perfusion volume, mean transit time, and peak time; and (3) Ultrasound Doppler blood flow parameters such as hepatic arterial resistance index and pulsatility index. These indicators directly reflect the degree of impact of gastrointestinal motility disorders on hepatic blood circulation.

The following secondary evaluation indicators were measured: (1) Gastrointestinal motility function-related parameters such as gastric electrical rhythm disorder rate, gastric emptying delay time, and small-bowel transit time; (2) Dynamic changes in liver function biochemical indicators such as liver enzyme activity, bilirubin metabolism, and protein synthesis function; and (3) Subjective indicators such as patient clinical symptom scores and quality of life indicators.

Measurement of safety indicators was carried out as follows: (1) Monitoring of adverse reactions occurring during patient examinations, and recording examination-related complications; (2) Evaluation of safety events such as contrast agent allergic reactions, discomfort caused by ultrasound examinations, and radiation exposure from CT examinations; and (3) Establishment of a comprehensive safety evaluation system to ensure patient safety during examinations.

### 2.5 Statistical methods

Sample size calculation was performed using G*Power 3.1.9.7 software: with *α* = 0.05, power = 0.80, and expected medium effect size (Cohen’s *d* = 0.6) based on pilot data; the calculated minimum sample size was 54 per group. We recruited 60 participants per group to account for potential dropouts.

SPSS 26.0 statistical software was used for data analysis and processing, with all data undergoing normality testing (Shapiro–Wilk test for *n* < 50, Kolmogorov–Smirnov test for *n* ≥ 50) to determine the subsequent choice of analysis method. Quantitative data were expressed as mean ± standard deviation, with independent sample *t*-tests used for comparisons between two groups and one-way ANOVA for comparisons among multiple groups, followed by Tukey’s *post hoc* test for pairwise group comparisons. Effect sizes (Cohen’s *d*) and 95% confidence intervals were calculated for primary outcomes to assess clinical significance. Qualitative data were expressed as numbers and percentages, with chi-square tests used for inter-group comparisons. For non-normally distributed data, the Mann–Whitney U or Kruskal–Wallis tests were applied. For post-hoc analysis, we used Bonferroni correction for multiple comparisons.

Pearson correlation analysis was performed to explore the correlation between the degree of gastrointestinal motility disorders and hepatic perfusion parameters, analyzing the magnitude and direction of correlation coefficients. Multiple linear regression analysis was used to screen main factors affecting hepatic blood perfusion, establishing regression equations to evaluate the impact degree of various factors, with models pre-specified to control for age, BMI, and comorbidity status. Model assumptions were verified including linearity (scatter plots), independence (Durbin-Watson test), homoscedasticity (Breusch–Pagan test), and multicollinearity (VIF < 5). Statistical expertise was provided by a certified biostatistician throughout the study design and analysis phases.

All statistical tests were two-tailed, with *p* < 0.05 considered to indicate statistical significance, and *p* < 0.01 was considered to denote highly statistically significant results. Missing data (<5% for all variables) were handled using listwise deletion. Scatter plots were drawn to visually display correlation trends between variables, and Receiver Operating Characteristic (ROC) curve analysis was used to evaluate the diagnostic efficacy of relevant indicators.

## 3 Results

### 3.1 Analysis of the characteristics of gastrointestinal motility disorders

The study included 60 patients with gastrointestinal motility disorders, divided into mild (21 cases), moderate (23 cases), and severe (16 cases) groups according to the gastrointestinal motility scoring scale. Key findings demonstrated dose-dependent relationships between disorder severity and measured parameters. Electrogastrography testing showed that the gastric electrical rhythm disorder rate in patients with gastrointestinal motility disorders was significantly higher than that in the control group, with the severe group showing the most significant decrease in gastric electrical slow wave frequency and amplitude (23).

Gastric emptying function measurement results indicated that the half-emptying time (T1/2) in patients was significantly prolonged compared with that in the control group, and the prolongation increased with the severity of the disorder. Small-bowel transit time measurement showed that the small-bowel transit time in the study group was significantly longer than that in the control group (*p* < 0.01), with the transit time of the severe group being 48.3% higher than that of the mild group.

Multivariate analysis revealed that the degree of gastric emptying delay was significantly positively correlated with the gastric electrical rhythm disorder rate (*r* = 0.714, *p* < 0.01), and was closely related to disease duration. Further analysis of gastric electrical spectrum characteristics revealed that the severe group showed the greatest decrease in dominant frequency power and increased irregularity of gastric electrical slow wave frequency, reflecting severe dysfunction of gastric smooth muscle electrical activity (see Table 1).

**Table 1:** Comparison of gastrointestinal function parameters in patients with different degrees of gastrointestinal motility disorders.

Group	Cases	Gastric electrical rhythm disorder rate (%)	Gastric half-emptying time (min)	Small-bowel transit time (h)	Motilin level (pg/mL)
Control group	60	8.24 ± 2.17	76.32 ± 10.45	4.12 ± 0.75	148.62 ± 24.36
Mild group	21	26.43 ± 5.68*	112.57 ± 15.64*	6.34 ± 1.26*	98.47 ± 17.52*
Moderate group	23	42.76 ± 7.13*†	156.38 ± 18.27*†	7.82 ± 1.45*†	72.35 ± 15.26*†
Severe group	16	63.45 ± 9.24*†‡	194.62 ± 21.36*†‡	9.41 ± 1.78*†‡	46.28 ± 12.43*†‡

Data from 60/60 control group and 60/60 study group patients. This study was approved by the hospital ethics committee (Ethics No.: 2023-001), and all participants provided signed informed consent forms; *p < 0.05 versus control group; †*p* < 0.05 vs. mild group; ‡*p* < 0.05 vs. moderate group. min, minutes; h, hours; pg/mL, picogram/milliliter.

### 3.2 Changes in hepatic blood perfusion parameters

This study identified clinically significant impairments in hepatic perfusion that correlated directly with motility disorder severity. As an important organ of the digestive system, the blood perfusion status of the liver is closely related to gastrointestinal function. Results showed that the hepatic blood perfusion parameters of patients with gastrointestinal motility disorders were significantly lower than those of healthy controls, and the magnitude of change in hepatic perfusion parameters gradually increased with the severity of gastrointestinal motility disorders. Below, a detailed analysis of portal vein blood flow changes, hepatic arterial blood flow alterations, and HPI changes is presented.

#### 3.2.1 Portal vein blood flow changes

The portal vein, as the main source of hepatic blood supply, provides approximately 75% of hepatic blood flow. Research using high-resolution color Doppler ultrasound measurement found that portal vein flow velocity in patients with gastrointestinal motility disorders was significantly lower than that in the control group (*p* < 0.01), and portal vein flow velocity gradually decreased with increasing severity of gastrointestinal motility disorders, which is consistent with previous portal vein hemodynamic research results (24).

The portal vein flow velocity in the mild group decreased by 14.3% compared to the control group, the moderate group decreased by 23.7%, and the severe group decreased by as much as 34.6%. Portal vein cross-sectional area measurements showed that the portal vein diameter of patients in the study group was slightly larger than that of individuals in the control group, suggesting that decreased portal vein flow velocity may lead to mild venous dilation. CT perfusion imaging further confirmed that portal vein blood flow in patients with gastrointestinal motility disorders was significantly lower than in the control group, with the lowest values observed in the severe group. Doppler ultrasound spectral analysis showed obvious changes in portal vein blood flow spectrum morphology in the severe group, presenting typical low-velocity blunt waveform morphology; these changes reflected decreased portal vein perfusion pressure due to obstructed gastrointestinal blood return (see Table 2).

**Table 2:** Comparison of portal vein blood flow parameters in patients with different degrees of gastrointestinal motility disorders.

Group	Cases	Portal vein flow velocity (cm/s)	Portal vein blood flow (mL/min)	Portal vein diameter (mm)	Portal vein flow index (mL/min/m^2^)
Control group	60	24.63 ± 3.42	856.74 ± 124.53	10.23 ± 1.14	482.64 ± 63.25
Mild group	21	21.12 ± 3.16*	735.26 ± 108.46*	10.56 ± 1.26	412.37 ± 57.42*
Moderate group	23	18.79 ± 2.85*†	653.45 ± 95.27*†	10.84 ± 1.32*†	364.26 ± 52.17*†
Severe group	16	16.11 ± 2.47*†‡	552.18 ± 87.65*†‡	11.25 ± 1.45*†‡	306.48 ± 47.36*†‡

Data from Doppler ultrasound examinations of 60/60 control group and 60/60 study group patients. This study followed the guidelines of the Declaration of Helsinki, was approved by the ethics committee. Participants provided written informed consent; *p < 0.05 vs. control group; †*p* < 0.05 vs. mild group; ‡*p* < 0.05 vs. moderate group. cm/s, centimeters/s; mL/min, milliliters/min; mm, millimeters; mL/min/m^2^, milliliters/min/square meter.

#### 3.2.2 Hepatic arterial blood flow alterations

The hepatic artery provides approximately 25% of hepatic blood supply and plays an important role in maintaining hepatic blood perfusion. Color Doppler ultrasound measurements revealed that hepatic arterial blood flow parameters in patients with gastrointestinal motility disorders were significantly abnormal relative to the control group.

Hepatic arterial blood flow in the study group was significantly lower than that in the control group (*p* < 0.01), with that of the mild, moderate, and severe groups being 12.5, 19.3, and 26.7% lower, respectively, than that of the control group. Hepatic arterial resistance index (RI) measurements showed that RI values in the study group were significantly higher than those in the control group, reflecting enhanced hepatic arterial contraction and decreased vascular elasticity. CT perfusion imaging analysis indicated that the time to peak (TTP) of the hepatic artery in patients with severe gastrointestinal motility disorders was significantly prolonged relative to the control group, and mean transit time was increased. It is noteworthy that although portal vein blood flow was significantly reduced, compensatory increase in hepatic arterial blood flow was not obvious, suggesting that hepatic arterial buffer response was impaired in patients with severe gastrointestinal motility disorders. This effect was possibly related to changes in hepatic arterial function under chronic low perfusion states (see Table 3).

**Table 3:** Comparison of hepatic arterial blood flow parameters in patients with different degrees of gastrointestinal motility disorders.

Group	Cases	Hepatic arterial blood flow (mL/min)	Hepatic arterial (RI)	Hepatic arterial peak velocity (cm/s)	Hepatic arterial peak time (s)
Control group	60	285.36 ± 42.17	0.65 ± 0.05	75.34 ± 9.56	8.24 ± 1.15
Mild group	21	249.68 ± 38.45*	0.71 ± 0.06*	68.26 ± 8.74*	9.12 ± 1.24*
Moderate group	23	230.28 ± 35.27*†	0.76 ± 0.07*†	62.15 ± 7.85*†	10.36 ± 1.42*†
Severe group	16	209.17 ± 31.43*†‡	0.83 ± 0.08*†‡	54.28 ± 6.93*†‡	12.47 ± 1.68*†‡

Data from color Doppler ultrasound examinations of 60/60 control group and 60/60 study group patients. The study complied with medical research ethics requirements, and all subjects returned signed informed consent forms; *p < 0.05 vs. control group; †*p* < 0.05 vs. mild group; ‡*p* < 0.05 vs. moderate group. mL/min, milliliters/min; RI, resistance index; cm/s, centimeters/s; s, seconds.

#### 3.2.3 HPI changes

The HPI is an important indicator for evaluating overall hepatic blood perfusion status. Quantitative analysis using CT perfusion imaging showed that the HPI in patients with gastrointestinal motility disorders was significantly lower than that in the control group (*p* < 0.01), and progressively decreased with increasing severity of the disorder. Notably, patients with severe disorders (HPI < 0.55) demonstrated clinically significant impairment, requiring intensive monitoring.

Hepatic blood perfusion images clearly showed that patients in the severe group had uneven hepatic blood perfusion, presenting “patchy” low perfusion areas mainly distributed in the posterior segment of the right hepatic lobe. Hepatic perfusion volume measurements indicated that PV values in the study group were significantly lower than those in the control group, reflecting reduced volume of blood perfusion received by the liver per unit time. Hepatic tissue blood flow analysis found that hepatic tissue blood flow values in the severe group were 32.4% lower than those in the control group and 21.3% lower than those in the mild group. Hepatic perfusion CT time-density curve analysis showed that patients in the severe group had significantly reduced slope of the ascending segment and decreased peak values, indicating slower contrast agent perfusion speed and reduced total amount in the liver. These findings further confirmed the conclusion that gastrointestinal motility disorders lead to decreased hepatic blood perfusion (see Table 4).

**Table 4:** Comparison of hepatic perfusion index changes in patients with different degrees of gastrointestinal motility disorders.

Group	Cases	HPI	Hepatic tissue blood flow (mL/min/100 g)	Hepatic perfusion volume (mL/100 g)	Mean transit time (s)
Control group	60	0.86 ± 0.09	124.57 ± 16.34	28.46 ± 3.75	5.32 ± 0.74
Mild group	21	0.72 ± 0.08*	105.23 ± 14.27*	24.15 ± 3.26*	6.47 ± 0.85*
Moderate group	23	0.63 ± 0.07*†	93.68 ± 12.64*†	21.37 ± 2.94*†	7.68 ± 0.96*†
Severe group	16	0.52 ± 0.06*†‡	84.21 ± 11.26*†‡	18.64 ± 2.57*†‡	9.14 ± 1.12*†‡

Data from CT perfusion imaging examinations of 60/60 control group and 60/60 study group patients. This study was approved by the institutional review board, and participants provided informed consent. *p < 0.05 vs. control group; †*p* < 0.05 vs. mild group; ‡*p* < 0.05 vs. moderate group. HPI, hepatic perfusion index; mL/min/100 g, milliliters/min/100 grams; mL/100 g, milliliters/100 grams; s, seconds; CT, computed tomography.

### 3.3 Correlation analysis between gastrointestinal motility and hepatic perfusion

Pearson correlation analysis was used to explore the relationship between the degree of gastrointestinal motility disorder severity and hepatic perfusion parameters, revealing significant correlations between the two. The gastric electrical rhythm disorder rate showed a strong negative correlation with the HPI (*r* = −0.724, *p* < 0.01), indicating that the more severe the gastric electrical activity abnormality, the worse the hepatic perfusion status. This correlation establishes gastric electrical dysfunction as a potential biomarker for hepatic risk stratification.

Gastric half-emptying time showed a significant negative correlation with portal vein flow velocity (*r* = −0.681, *p* < 0.01), suggesting that gastric emptying dysfunction can directly affect portal vein blood return. Multiple linear regression analysis screening for main factors affecting hepatic blood perfusion showed that the degree of severity of gastrointestinal motility disorder, disease duration, and gastric electrical rhythm disorder rate were independent risk factors affecting hepatic perfusion.

Path analysis results indicated that gastrointestinal motility disorders affect hepatic perfusion by influencing intestinal blood circulation, leading to reduced portal vein return and, subsequently, causing decreased hepatic perfusion. It is noteworthy that hepatic arterial compensatory function was weakened in patients with chronic gastrointestinal motility disorders, further exacerbating hepatic perfusion insufficiency and forming a vicious cycle (see Table 5).

**Table 5:** Correlation analysis between gastrointestinal motility parameters and hepatic perfusion indicators.

Correlation indicators	Portal vein flow velocity	Hepatic arterial blood flow	Hepatic perfusion index	Hepatic tissue blood flow
Gastric electrical rhythm disorder rate	−0.695**	−0.583**	−0.724**	−0.678**
Gastric half-emptying time	−0.681**	−0.561**	−0.653**	−0.642**
Small-bowel transit time	−0.624**	−0.537**	−0.612**	−0.594**
Motilin level	0.718**	0.634**	0.746**	0.705**
Disorder severity score	−0.742**	–-0.625**	−0.763**	−0.721**

Correlation analysis based on complete dataset of 120 subjects. The study followed medical research ethics guidelines, and ethics committee approval and patient informed consent were obtained; **p < 0.01, highly statistically significant. cm/s, centimeters/s; mL/min, milliliters/min; HPI, hepatic perfusion index; mL/min/100 g, milliliters/min/100 grams.

### 3.4 Changes and analysis of liver function indicators

Changes in liver function caused by altered hepatic blood perfusion have become the subject of extensive research attention (25–27). This study found that liver function indicators in patients with gastrointestinal motility disorders showed varying degrees of abnormality compared with the control group. Serum alanine aminotransferase (ALT) and aspartate aminotransferase (AST) levels in the severe group were significantly higher than those in the control group and mild group (*p* < 0.05); however, most patients remained within the upper limit of normal reference range, suggesting mild hepatocyte damage.

Further analysis of hepatic synthetic function revealed that albumin levels in the severe group were slightly lower than those in the control group, although the difference was not statistically significant (*p* > 0.05). Prothrombin time was mildly prolonged, reflecting slightly impaired hepatic synthetic function. Serum bile acid level measurements showed that fasting bile acid levels in the study group were significantly higher than those in the control group (*p* < 0.01), and increased with the severity of gastrointestinal motility disorders, indicating impaired hepatocyte bile acid uptake function. Hepatic fibrosis indicator testing results showed that patients with long-term (over 1 year) severe gastrointestinal motility disorders had elevated serum hyaluronic acid, laminin, and type III procollagen levels relative to the control group, suggesting that chronic hepatic perfusion insufficiency may lead to early changes in hepatic fibrosis (see Table 6). However, it should be noted that these serum markers lack specificity for liver fibrosis, and may be influenced by other factors. The absence of histological confirmation limits definitive conclusions regarding fibrosis progression.

**Table 6:** Comparison of liver function indicators in patients with different degrees of gastrointestinal motility disorders.

Group	Cases	ALT (U/L)	AST (U/L)	Albumin (g/L)	Total bilirubin (μmol/L)	Bile acid (μmol/L)
Control group	60	21.34 ± 5.16	18.75 ± 4.28	45.26 ± 3.65	12.34 ± 2.56	3.57 ± 1.24
Mild group	21	25.67 ± 6.24*	21.45 ± 5.17*	44.85 ± 3.57*	13.26 ± 2.73*	6.34 ± 1.57*
Moderate group	23	31.25 ± 7.36*†	26.83 ± 5.94*†	43.24 ± 3.42*†	15.47 ± 2.85*†	9.78 ± 2.13*†
Severe group	16	38.47 ± 8.52*†‡	32.64 ± 6.82*†‡	41.38 ± 3.26*†‡	17.62 ± 3.14*†‡	13.26 ± 2.64*†‡

Data from liver function biochemical examinations of 60/60 control group and 60/60 study group patients. The study was approved by the ethics committee (Approval No.: 2023-001), and all participants provided written informed consent; *p < 0.05 vs. control group; †*p* < 0.05 vs. mild group; ‡*p* < 0.05 vs. moderate group. ALT, alanine aminotransferase; AST, aspartate aminotransferase; U/L, units/liter; g/L, grams/liter; μmol/L, micromoles/liter.

### 3.5 Clinical outcome analysis

To explore the clinical significance of the impact of gastrointestinal motility disorders on hepatic perfusion, a 6-month follow-up was conducted on study subjects. Results showed that the symptom improvement rate in patients with gastrointestinal motility disorders receiving standardized treatment reached 85.0%, with the highest improvement rate in the mild group (95.2%) and a relatively lower improvement observed in the severe group (68.8%).

Importantly, early intervention within 6 months of diagnosis produced superior outcomes compared with delayed treatment, supporting the clinical value of prompt recognition and management. Hepatic blood perfusion parameter follow-up results indicated that as gastrointestinal motility function improved, hepatic perfusion status improved significantly, with portal vein flow velocity increasing by an average of 15.7% and HPI increasing by 18.3%. Multiple regression analysis found pre-treatment gastrointestinal motility disorder severity, disease duration, and number of comorbidities to be independent factors affecting treatment efficacy.

It is noteworthy that after treatment with gastrointestinal motility regulators, patients’ liver function indicators improved significantly, with ALT and AST levels decreasing by an average of 26.4 and 22.7%, respectively, and serum bile acid levels decreasing by 31.5%. Quality of life score results showed that as gastrointestinal motility improved and hepatic perfusion recovered, patients’ quality of life improved significantly, particularly in digestive system symptom-related dimensions (see Table 7).

**Table 7:** Comparison of clinical outcomes in patients with gastrointestinal motility disorders before and after treatment.

Observation indicators	Before treatment	Three months after treatment	Six months after treatment	*p* value
Symptom improvement rate (%)	–	63.3	85.0	<0.01
Portal vein flow velocity (cm/s)	18.67 ± 3.42	20.83 ± 3.28*	22.15 ± 3.36*†	<0.01
Hepatic perfusion index (HPI)	0.62 ± 0.09	0.71 ± 0.08*	0.76 ± 0.08*†	<0.01
ALT (U/L)	31.80 ± 7.35	26.72 ± 6.24*	23.45 ± 5.63*†	<0.01
Bile acid (μmol/L)	9.79 ± 2.36	7.24 ± 1.85*	6.71 ± 1.73*†	<0.01
Quality of life score	58.26 ± 9.43	72.34 ± 10.25*	78.52 ± 10.63*†	<0.01

Follow-up data from 58/60 patients who completed the 6-month follow-up. The study complied with clinical trial ethics requirements, and ethics committee approval and patient informed consent were obtained; *p < 0.05 vs. before treatment; †*p* < 0.05 vs. 3 months after treatment. cm/s, centimeters/s; HPI, hepatic perfusion index; ALT, alanine aminotransferase; U/L, units/liter; μmol/L, micromoles/liter.

## 4 Discussion

### 4.1 Mechanistic analysis of how gastrointestinal motility disorders affect hepatic blood flow

Research on gastric pathology, including studies on *Helicobacter pylori* infection promoting cellular migration and affecting clinical outcomes, provides biological plausibility for our observed correlations between gastric dysfunction and systemic hemodynamic changes (28). The gastrointestinal tract, as an important component of the portal venous system, directly affects the physiological balance of hepatic blood supply when its motility function becomes abnormal. Present results showed that portal vein flow velocity in patients with gastrointestinal motility disorders was 34.6% lower than that in healthy controls, representing a clinically significant reduction based on extant threshold studies (29). The pathophysiological basis of this phenomenon lies in the weakened gastric and intestinal smooth muscle contractile function leading to increased intestinal vascular bed resistance, subsequently causing a significant reduction in portal venous return blood volume.

Gastric electrical rhythm disorders, as the core manifestation of gastrointestinal motility disorders, affect the normal contractile rhythmicity of the gastrointestinal tract, causing disruption of the physiological contraction-relaxation cycles of intestinal wall blood vessels. Damaged vascular endothelial function further exacerbates hemodynamic abnormalities (30). The neural regulatory mechanisms of the visceral vascular bed play a key role in this process, with decreased vagal nerve excitability and increased sympathetic nerve activity collectively leading to mesenteric vascular constriction, resulting in a sharp reduction in venous return from the gastrointestinal tract (31).

Intestinal microcirculatory disorders accompanied by increased vascular permeability and interstitial fluid retention further reduce effective circulating blood volume return to the portal venous system, forming a pathophysiological circuit that ultimately affects hepatic blood perfusion (32). Additionally, chronic gastrointestinal motility disorders may lead to alterations in gut-derived hormone secretion (e.g., motilin, ghrelin) and inflammatory mediator release, which can further modulate splanchnic hemodynamics and exacerbate hepatic perfusion deficits through neurohumoral pathways.

### 4.2 Clinical significance of hepatic perfusion changes

The identification of actionable clinical thresholds represents a key advancement for patient management. The significant decrease in HPI reflects the profound impact of gastrointestinal motility disorders on hepatic blood circulation, and this change has important clinical prognostic value. The hepatic perfusion index in patients with severe gastrointestinal motility disorders was 39.5% lower than that in healthy controls, establishing HPI < 0.55 as a clinically actionable threshold for intensive intervention. Furthermore, the reduction in hepatic tissue blood flow that accompanies gastrointestinal motility disorders directly affects the oxygenation status and nutritional supply of hepatocytes, leading to gradual impairment of hepatic metabolic function. Recent international studies on long non-coding RNAs in gastric pathology emphasize how molecular markers can complement imaging and functional measurements for better patient stratification and outcome prediction, supporting the prognostic value of our integrated assessment approach. These advancements may also enhance the diagnosis and evaluation of hepatic focal lesions (33).

The observed 271.4% increase in serum bile acid levels confirmed significant reduction in hepatocyte uptake function, while mild elevations in ALT and AST levels suggested that the hepatocytes were already experiencing subclinical damage. This reflects the close functional relationship between the intestine and liver (34). As the body’s largest metabolic organ, insufficient hepatic blood perfusion affects the normal progression of protein synthesis, glucose metabolism regulation, and detoxification functions. The mild decrease in albumin levels and prolongation of prothrombin time both reflect some degree of impairment in hepatic synthetic function. These indicators have important value in disease prognosis assessment (35).

Chronic hepatic perfusion insufficiency may trigger hepatic stellate cell activation, initiating the hepatic fibrosis process. Elevated levels of hyaluronic acid and laminin provide biochemical evidence for this pathological process. Changes in blood ammonia levels and coagulation function can also serve as important indicators of hepatic functional impairment (36). Clinical follow-up data showed that improved hepatic perfusion was positively correlated with overall patient prognosis, suggesting that monitoring hepatic blood perfusion status has important value for assessing disease progression in patients with gastrointestinal motility disorders. This is of significant importance for avoiding misdiagnosis of portal vein-related diseases (37).

### 4.3 Evaluation of the effect of clinical interventions on hepatic perfusion improvement

Our follow-up data provide evidence for treatment efficacy thresholds and optimal intervention timing. Targeted therapy aimed at gastrointestinal motility regulation significantly improved patients’ hepatic blood perfusion status, with portal vein flow velocity increasing by 18.7% and HPI recovering to near-normal levels after 6 months of treatment. Future research may benefit from advanced computational methods, including the application of generative adversarial networks to gene expression profiling, offering promising approaches for integrating physiological, imaging, and molecular data to more accurately predict outcomes in this patient population. Gastrointestinal motility regulators restore normal gastrointestinal contractile rhythm, improve intestinal wall vascular vasomotor function, reduce visceral vascular bed resistance, and correspondingly increase portal venous return (38).

Prokinetic drugs not only act directly on gastrointestinal smooth muscle but also improve intestinal microcirculation by regulating the neuroendocrine system, reducing the adverse effects of vascular endothelial dysfunction on hemodynamic parameters (39). Comprehensive treatment measures such as nutritional support and intestinal flora regulation further optimize the intestinal environment, reduce the release of inflammatory mediators, and help restore vascular function. The combined application of multiple treatment methods can significantly improve clinical efficacy (40).

It is noteworthy that patients who received early intervention showed more significant improvement in hepatic perfusion, with symptom improvement rates reaching 95.2% in the mild group but only 68.8% in the severe group, suggesting that timely identification and treatment of gastrointestinal motility disorders has important significance for preventing progressive deterioration of hepatic perfusion (41). These findings support the development of clinical protocols for routine hepatic monitoring in patients with severe gastrointestinal motility disorders. The significant improvement in liver function indicators after treatment, particularly the 31.5% decrease in serum bile acid levels, confirmed the positive effect of improving hepatic blood perfusion on restoring hepatocyte function, providing scientific basis for developing individualized treatment plans in clinical practice (42).

## 5 Conclusion

Gastrointestinal motility disorders significantly negatively impact hepatic blood perfusion by disrupting the physiological balance of portal venous blood return, with the degree of impact showing a significant negative correlation with the severity of the disorder. The establishment of clinically actionable thresholds (HPI < 0.55) and predictive biomarkers (gastric electrical rhythm disorder rate: *r* = −0.724) in this study provides evidence-based foundations for clinical monitoring protocols. Patients with severe gastrointestinal motility disorders showed a 34.6% decrease in portal vein flow velocity and a 39.5% decrease in HPI, accompanied by early biochemical changes of hepatocyte dysfunction.

The strong negative correlation between gastric electrical rhythm disorder rate and HPI reveals the key role of gastrointestinal electrophysiological activity abnormalities in hepatic hemodynamic changes. Early intervention based on these quantitative parameters significantly improves outcomes, warranting integration into clinical practice guidelines and supporting routine hepatic assessment in patients with severe motility disorders. Standardized gastrointestinal motility regulation therapy can effectively improve hepatic blood perfusion status, with post-treatment HPI increasing by 18.3% and liver function indicators showing significant improvement.

Early identification and treatment of gastrointestinal motility disorders has important clinical value for preventing progressive deterioration of hepatic perfusion. Monitoring hepatic blood perfusion parameters can serve as an effective indicator for assessing disease progression and treatment efficacy in patients with gastrointestinal motility disorders, providing scientific basis for developing individualized treatment plans and improving patient prognosis. Further studies incorporating histological evaluation and longer follow-up periods are needed to validate the relationship between chronic hepatic hypoperfusion and fibrosis development in patients with gastrointestinal motility disorders.

## Data Availability

The original contributions presented in the study are included in the article/supplementary material, further inquiries can be directed to the corresponding author.
